# Effect of Immobilized Antithrombin III on the Thromboresistance of Polycarbonate Urethane

**DOI:** 10.3390/ma10040335

**Published:** 2017-03-24

**Authors:** Karin Lukas, Karin Stadtherr, Andre Gessner, Daniel Wehner, Thomas Schmid, Hans Peter Wendel, Christof Schmid, Karla Lehle

**Affiliations:** 1 IMHR, Institute for Medical Microbiology and Hygiene, University of Regensburg, Franz-Josef-Strauss-Allee 11, 93042 Regensburg, Germany; Karin.Lukas@UKR.de (K.L.); Karin.Stadtherr@gmail.com (K.S.); Andre.Gessner@UKR.de (A.G.); 2 Dualis Medtech GmbH, Am Technologiepark 8+10, 82229 Seefeld, Germany; Daniel.Wehner@dualis-medtech.de (D.W.); Thomas.Schmid@hotmail.de (T.S.); 3 Department of Thoracic, Cardiac, and Vascular Surgery, University Hospital Tübingen, Hoppe-Seyler-Str. 3, 72076 Tübingen, Germany; Hans-Peter.Wendel@med.uni-tuebingen.de; 4 Department of Cardiothoracic Surgery, University Medical Center Regensburg, Josef-Strauss-Allee 11, 93042 Regensburg, Germany; Christof.Schmid@UKR.de

**Keywords:** polycarbonate urethane, thromboresistance, antithrombin III, anti-bacterial, hemocomaptibility

## Abstract

The surface of foils and vascular grafts made from a thermoplastic polycarbonate urethanes (PCU) (Chronoflex AR) were chemically modified using gas plasma treatment, binding of hydrogels—(1) polyethylene glycol bisdiamine and carboxymethyl dextran (PEG-DEX) and (2) polyethyleneimine (PEI)—and immobilization of human antithrombin III (AT). Their biological impact was tested in vitro under static and dynamic conditions. Static test methods showed a significantly reduced adhesion of endothelial cells, platelets, and bacteria, compared to untreated PCU. Modified PCU grafts were circulated in a Chandler-Loop model for 90 min at 37 °C with human blood. Before and after circulation, parameters of the hemostatic system (coagulation, platelets, complement, and leukocyte activation) were analyzed. PEI-AT significantly inhibited the activation of both coagulation and platelets and prevented the activation of leukocytes and complement. In conclusion, both modifications significantly reduce coagulation activation, but only PEI-AT creates anti-bacterial and anti-thrombogenic functionality.

## 1. Introduction

Polycarbonate urethanes (PCUs) have been used in implantable medical devices because of their superior biocompatibility, attractive mechanical properties, and improved oxidative biostability [1,2,3,4,5]. However, thrombus formation may still occur when artificial organs and biomedical devices made of PCU are in contact with blood for extended periods of time [6,7,8]. Currently this is managed by anti-platelet therapy, however, this presents a risk of bleeding [8]. Therefore, there remains a pressing need for non-thrombogenic surface coatings that can reduce this reliance on pharmacological intervention. Approved surface coatings include the immobilization of drugs that block fibrin formation and prevent platelet adhesion/activation. Promising coating strategies for blood contacting medical devices are the immobilization of heparin [9,10,11], thrombomodulin [12], phosphatidylcholine [13], and argatroban [14,15].

The present study pursued the strategy to immobilize antithrombin III (AT) on the surface of a plasma-treated thermoplastic PCU (Chronoflex AR) that was linked by polyethylene glycol bisdiamine (PEG), carboxymethylied dextran (DEX), and polyethylenimine (PEI)-based hydrogels. AT is a serine protease inhibitor that inactivates proteases from the contact activation pathway, mainly activated factor II (IIa, thrombin). However, contact of blood with polymeric surfaces can result in a decrease in the AT activity as shown during cardiopulmonary bypass operation [16] or after the implantation of mechanical support devices such as ventricular assist devices [17]. The reduction in AT activity can be responsible for subclinical coagulation, heparin resistance, and clinically relevant thromboembolic events [6]. The application of recombinant AT may be one method of avoiding these complications in heparin-resistant patients that require cardiopulmonary bypass operation [18]. Furthermore, Sask and coworkers immobilized an AT-heparin anticoagulant complex on the surface of a segmented polyurethane and demonstrated improved anticoagulant properties for this surface via direct thrombin inhibition by the AT portion of the complex [19].

In this work, human AT was used to modify the surface of a commercially available PCU linked with different hydrogels (PEG-DEX and PEI). In a previous study we showed that covalent binding of AT improved anti-thrombotic properties of untreated PCU, independent of the type of connecting hydrogel [20]. However, reproducible low platelet adhesion was only documented for the combination of PEG-DEX and PEI with AT [20]. Therefore, these modifications were analyzed to verify their antithrombotic functionality under physiological conditions, such as shear stress. The hemocompatibility of the two surface modifications was tested according to ISO 10993-4 using a Chandler-Loop model in order to exclude hemostatic activation induced by the surface chemistry [21].

## 2. Methods

### 2.1. Production of PCU Foils and Spiral PCU Grafts

The aromatic PCU ChronoFlex AR (CF, AdvanSource Biomaterials, Wilmington, MA, USA) was processed according to the manufacturer’s instructions [1].

Processing of foils: Dissolved CF (12 mL) was poured into a flat mold (155 mm × 25 mm × 5 mm) and dried in a vented air oven (Carbolite, Neuhausen, Germany) (45 °C, 12 h). The foil (thickness, 0.5 mm) was incubated in a heated vacuum chamber (Binder, Tuttlingen, Germany) (1.5 mbar, 75 °C, 3 days). Round (diameter, 6 mm) or rectangular samples (12 mm × 3.5 mm) were prepared.

Processing of spiral PCU grafts: A helically wound, round stock made of aluminum (diameter, 5 mm; length, 600 mm) was mounted onto a robot (Kuka, Augsburg, Germany). The stock was dipped into dissolved CF, and the robot panned the stock (around its helical and horizontal axis) in a climate chamber (custom creation; relative humidity ≤30%; 28 °C). The dipping process was repeated three times to obtain a wall thickness of 0.5 mm. After having dried in the climate chamber, the helical graft was placed into the vented air oven (45 °C, 12 h), incubated in the heated vacuum chamber, and immersed in ethanol (≥99.5%, p.a., Carl Roth, Karlsruhe, Germany) (room temperature, RT; 6 h) to facilitate the removal of the graft from the stock.

### 2.2. Surface Modifications

The details on handling and modifying the PCU discs and helical grafts have been described previously [20]. Chemicals were obtained from Merck (Darmstadt, Germany) or Sigma-Aldrich (Munich, Germany), unless noted otherwise.

Material was cleaned briefly in ethanol, dried, and activated by modified N_2_ plasma treatment with an additional supply of carbon dioxide. Plasma-activated PCU (pPCU) was incubated in 0.9% sodium chloride solution (NaCl) (50 °C, 2 h), and incubated in aqueous NHS/EDC (*N*-hydroxysuccinimide/1-ethyl-3-(3-dimethylaminopropyl) carbodiimide) (0.1 M/0.1 M) (RT, 20 min) (activated PCU samples).

PEG-DEX-AT modification: Activated PCU samples were incubated in 0.1 M carbonate buffer pH 8.4/0.6 M potassium sulfate with 1 mg/mL polyethylene glycol bisamine (PEG-diamine; Sigma-Aldrich) (50 °C, 2 h) [22] (PEG). After having been cleaned with deionized water, carboxymethyl dextran (DEX) was immobilized at the activated sample surfaces (PEG-DEX). To accomplish immobilization, DEX synthesized by the reaction of dextran (Sigma-Aldrich) with bromoacetic acid [23] was incubated in aqueous EDC/NHS (0.2 M/0.1 M) for 20 min. PEG samples were coupled with 1 mg/mL of activated DEX in carbonate buffer environment (2 h, RT) (PEG-DEX). Having been rinsed in water, PEG-DEX was treated with aqueous EDC/NHS (0.1 M/0.1 M) and incubated overnight with 1 IU/mL of human antithrombin III (AT; Kybernin^®^ P, CLS Behring, Marburg, Germany) at 4 °C.

PEI-AT modification: pPCU samples were incubated with 3 mg/mL branched polyethyleneimine (PEI, Sigma-Aldirch) (2 h, RT) (PEI). AT was preactivated in aqueous EDC/NHS (0.2 M/0.1 M) (20 min, RT). Subsequently, preactivated AT (1 IU/mL) was incubated with PEI samples (19 h, 4 °C) (PEI-AT). All modified samples were washed with deionized water, dried, and stored at 4 °C.

Untreated and modified PCU samples were blinded by the allocation of numbers.

### 2.3. Elasticity and Wettability of Surface Modifications

The focus of the present study is the analysis of the biological effect of surface modifications. Therefore, only a few methods were performed to describe surface characteristics: the maintenance of elasticity (Dynamical Mechanical Analysis, DMA) and an examination of the wettability. Fourier transform infrared (FTIR) and nuclear magnetic resonance (NMR) spectroscopy were not performed.

DMA was used to analyze the temperature-dependent visco-elastic properties and to determine the complex modulus (E′, modulus of elasticity; E″, damping values; tan d = E″/E′) using Netzsch-DMA 242 C (Netzsch-Gerätebau, Selb, Germany). After applying sinusoidal stress (7 N, 1 Hz) to the sample, we measured the strain in the material while changing the temperature (−100 °C to 100 °C; heating rate, 2 K/min). In this way, we determined the change in the glass transition temperature of the rectangular foil materials that was caused by surface modification.

Wettability was evaluated by means of water contact angles using the sessile drop. A drop of deionized water (2 µL) was put onto the sample surface. We photographed the drops after exactly 10 s and measured the contact angle values [24]. Each sample was measured five times, and the results were averaged.

### 2.4. Bacterial Adhesion

Bacterial adhesion assay: Bacterial adhesion was initiated with gram-positive Staphylococcus epidermidis. *S. epidermidis* is a common human commensal microorganism that colonizes skin and mucosal surfaces; it has become an opportunistic pathogen, due to its ability to colonize invasive medical devices causing blood stream infections [25]. Only untreated, PEG-DEX-AT and PEI-AT samples were analyzed. Untreated and modified PCU discs were inserted into 96-well polypropylene microplates (Nunc, Thermo Fisher, Waltham, MA, USA) and incubated in a suspension of bacteria (100 µL; 12 × 10^5^ colony forming unit, CFU mL^−1^) in BM medium (tryptone, 10 g/L; yeast extract, 5 g/L; NaCl, 5 g/L; K_2_HPO_4_, 1 g/L; glucose, 1 g/L) (37 °C, 30 min). The samples were carefully rinsed in sterile isotonic phosphate buffer saline (PBS) under continuous shaking at 100 rpm. The remaining solution was blown off with sterile, compressed air. The surface of the discs was placed on blood agar plates (37 °C, 19 h). Colony-forming bacteria produced recesses in the agar surface that were counted per surface area of the disc. The ratio of bacteria adherent to modified PCU discs compared to untreated PCU samples was analyzed.

Bacterial proliferation assay: Untreated and modified PCU discs were incubated with *S. epidermidis* for 8 h and 120 h, gently washed, fixed with pure methanol, stained with crystal violet (0.2% aqueous solution; 15 min), and rinsed with PBS. We extracted the crystal violet that was bound to the bacteria with 200 µL ethanol and determined optical density at 595 nm.

### 2.5. Platelet Adhesion—Hemocompatibility

In a previous study, a static platelet adhesion test was used to discriminate the platelet adhesion of different surface modifications, including all intermediate modified PCU materials [20]. In brief, plasma-activation did not improve hemocompatibility. The crucial step was the binding of PEG to significantly reduce platelet adhesion. Subsequent binding of DEX and AT showed no additional improvement. In contrast, the binding of PEI alone did not affect platelet adhesion. The additional coupling of AT induced a significant increase in thromboresistance of PCU. The same test was used to emphasize the extent of platelet adhesion on PEG-DEX-ATT and PEI-ATT modified PCU samples using scanning electron microscopy (SEM). Human citrated venous blood was drawn from healthy male volunteers who had given their written consent, as per institutional ethics guidelines (No. 10-101-0159). Platelet-rich plasma (PRP) was isolated as described earlier [20]. For platelet adhesion testing, PRP (50 µL; 5 × 10^7^ platelets per 0.3 cm^2^) was coincubated with the PCU samples (37 °C, 5% CO_2_, 30 min), washed with PBS and processed as described in Section 2.7.

### 2.6. Chandler-Loop Experiments

In vitro experiments were conducted with a dynamic rotation model (modified Chandler-Loop) [26]. The experiments were done in the laboratory of the Department of Throacic and Cardiovascular Surgery at University Hospital Tuebingen. Each spiral PCU graft (untreated and treated) was filled with 12 mL of donor blood. The loops were vertically rotated (37 °C, 90 min). Blood was collected for cell counting and measurement of complement (SC5b-9), thrombin-antithrombin-complex (TAT), polymorphonuclear (PMN)-elastases, and ß-thromboglobulin (TG) levels. Blood without any contact to material was used as negative control. Changes in markers of coagulation, complement activation, and blood cell release factors were measured using commercially available enzyme-linked immunosorbent assay (ELISA) kits (TG (Asserachrom ß-TG, Diagnostica Stago, Asnieres, France); TAT (Enzygnost TAT micro, Dade Behring, Schwalbach, Germany); PMN-elastase (Demeditec, Diagnotics, Kiel, Germany); SC5b-9 (Quidel, San Diego, CA, USA)). Blood cells were counted in EDTA blood using a fully automated cell counter system (micros 60 ABX Hematology, Montpellier, France).

### 2.7. Scanning Electron Microscopy (SEM)

Thrombogenicity of the modified spiral grafts and flat discs was verified with SEM. After the end of the experiments, the samples were rinsed with PBS and incubated in 2% glutaraldehyde. After extensive rinsing with PBS, the remaining water was then removed from the grafts using 40%–100% ethanol in ascending concentrations. Finally, all samples underwent critical point drying, were sputtered with gold palladium, and were subsequently analyzed with SEM (EVO-LS10, Zeiss, Oberkochen, Germany).

### 2.8. Statistics

Data are presented as median (IQR, interquartile range) and were analyzed with the Wilcoxon Signed Rank Test (Sigma-Stat, SPSS, Chicago, IL, USA) after having passed the Friedman Test (Sigma-Stat, SPSS). *p* values of ≤0.05 were considered significant.

## 3. Results

### 3.1. Mechanical Properties

**Figure 1:**
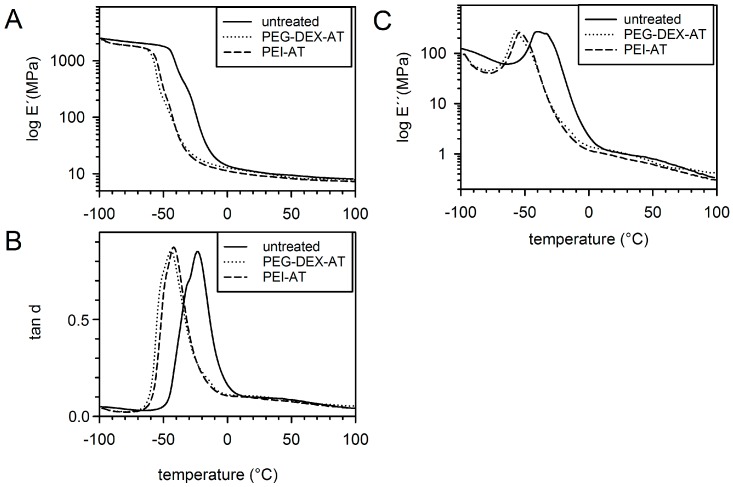
Mechanical properties of untreated and modified polycarbonate urethanes (PCU) samples. DMA curves of (**A**) elastic modulus (E′) and (**B**) tan d; (**C**) loss modulus curves (E″) versus temperature for untreated and modified PCU samples.

The dynamic mechanical relaxation behavior of the rectangular untreated and modified PCU samples is shown in Figure 1. The glass transition of the analyzed samples was identified by the respective drops in the storage modulus (E′) and prominent peaks in tan d and loss modulus (E″). The glass transition temperature of the untreated PCU sample was higher than that of any modified PCU sample (untreated, −24 °C; PUR-DEX-A, −44 °C; PEI-AT, −42 °C). This difference in glass transition temperatures was attributed to a greater flexibility of the modified PCU samples than that of the untreated PCU samples. The different surface modifications did not differ with regard to their elastic properties. At 37 °C, the measured values were within the same range, which indicated the absence of a significant difference in the material properties between untreated and modified samples (representative data for E’ (Pa): untreated, 9.82; PUR-DEX-A, 9.59; PEI-AT, 8.77; data for tan d: untreated, 0.094; PUR-DEX-A, 0.093; PEI-AT, 0.083).

### 3.2. Wettability

The contact angles of water on AT-modified PCU discs (PEG-DEX-AT, 62° ± 3°; PEI-AT, 58° ± 2.6°) were significantly reduced compared to untreated PCU samples (78° ± 1.7°; *p* < 0.001 vs. each modification). There was no difference in the contact angles of the AT-modified samples (*p* = 0.081).

### 3.3. Biofilm Formation on Modified PCU

**Figure 2:**
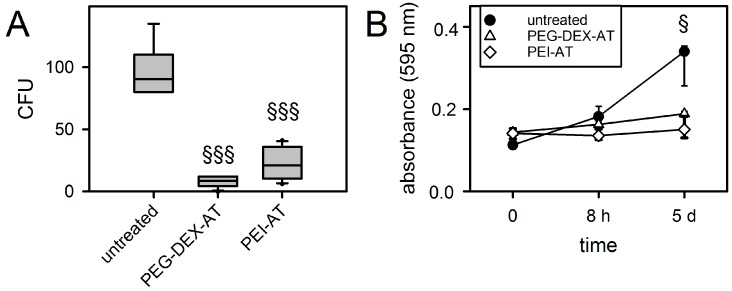
Bacterial adhesion and biofilm formation of untreated and modified PCU samples. Each modification significantly reduced bacterial adhesion (*n* = 12) (**A**); and biofilm formation (*n* = 3) (**B**); compared to untreated PCU (§, *p* ≤ 0.05; §§§, *p* ≤ 0.001). CFU, colony forming unit. Data are presented as median with 25/75 and 5/95 percentile (error bars).

Bacterial adhesion and proliferation on the surface of biomaterials is essential for biofilm formation. The amount of adhering *S. epidermidis* was highest on untreated PCU samples. As shown in Figure 2A, CFUs were significantly reduced on PEG-DEX-AT (8% ± 4%) and PEI-AT (23% ± 13%), compared to untreated PCU (each, *p* ≤ 0.001). In addition, bacterial proliferation was inhibited after contact with all modified PCU samples. Only untreated PCU samples showed a significant increase in biofilm formation after 5 days (*p* = 0.013; Figure 2B).

### 3.4. Hemocompatibility under Static Culture Conditions

**Figure 3:**
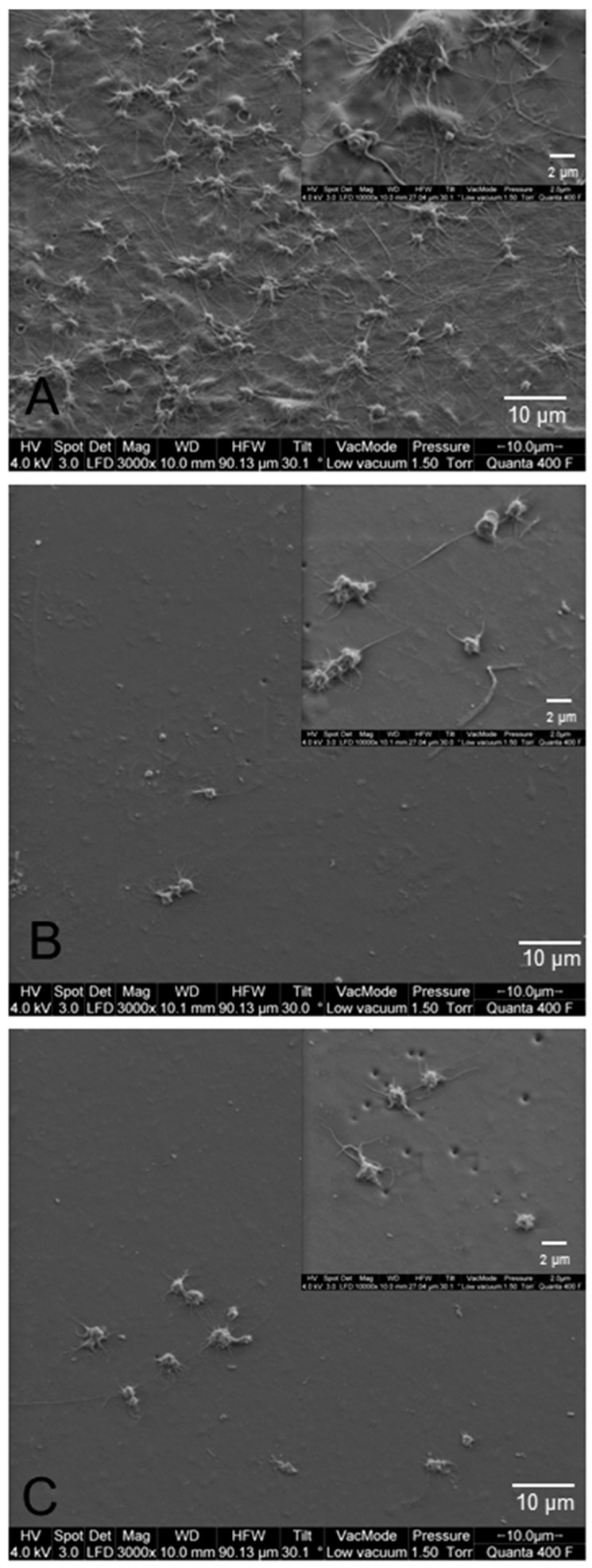
Hemocompatibility using a static platelet adhesion assay. Representative samples of (**A**) untreated PCU; (**B**) polyethylene glycol bisdiamine–carboxymethyl dextran–antithrombin III (PEG-DEX-AT); and (**C**) polyethyleneimine (PEI)-AT modified PCU. The samples were incubated with platelet-rich plasma. Adherent platelets were visualized with scanning electron microscopy (SEM). Inserts present higher magnification. The test was replicated with 5 platelet donors (independent experiments).

The surface of untreated PCU samples was densely covered with fibrin networks and activated platelets (Figure 3A). The activated platelets showed spread platelets with long pseudopodia. In contrast, few platelet deposits were found on PEG-DEX-AT- and PEI-AT-modified surfaces (Figure 3B,C). Single platelets with few and short pseudopodia were visible.

### 3.5. Chandler-Loop Experiments

**Figure 4:**
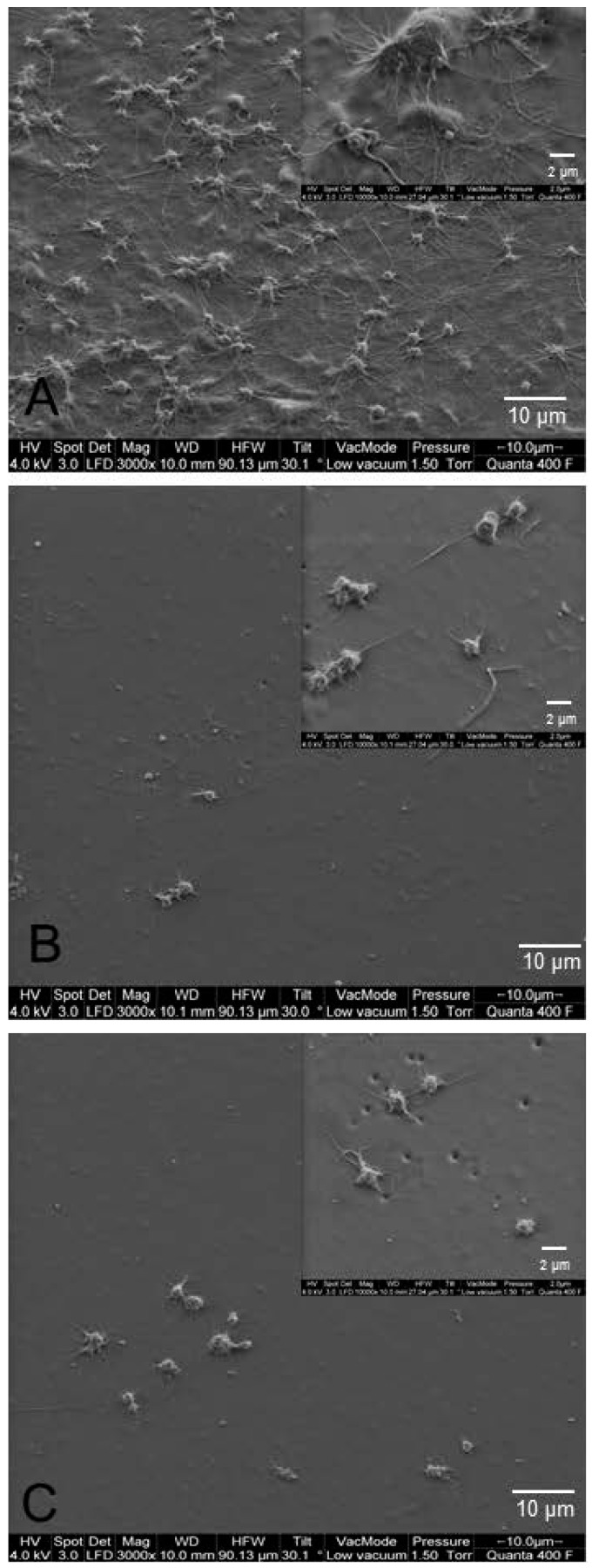
Platelet adhesion after recirculation of heparinized blood in grafts made of (**A**) untreated PCU; (**B**) PEG-DEX-AT; and (**C**) PEI-AT in the Chandler-Loop model. White arrow indicates adherent platelets in representative images of scanning electron microscopy (SEM). The test was replicated with 5 blood donors (independent experiments).

No material showed any sign of clot formation after 90 min recirculation in the Chandler-Loop model. SEM showed more platelet sticking and adherence of blood cell on the surface of untreated PCU samples (Figure 4).

**Figure 5:**
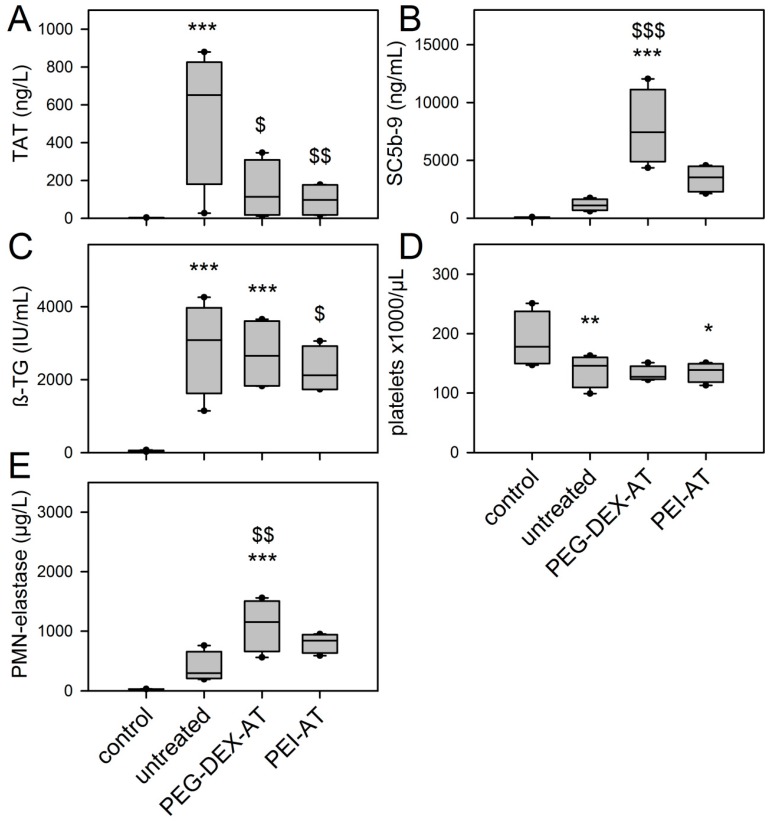
Effect of untreated and modified PCU samples on blood responses using a Chandler-Loop model. Specific parameters were detected after 90 min (**A**) Thrombin-antithrombin III complex (TAT); (**B**) SC5b-9; (**C**) ß-thromboglobulin (ß-TG); (**D**) platelet count; and (**E**) PMN-elastase (PMN, polymorphonuclear). Data from 5 independent runs are presented as median with 25/75 and 5/95 percentile (error bars). Statisitcs: *, significant alteration compared to control (*, *p* ≤ 0.05; **, *p* ≤ 0.05; ***, *p* ≤ 0.001); $, significant difference compared to untreated PCU samples ($, *p* ≤ 0.05; $$, *p* ≤ 0.05; $$$, *p* ≤ 0.001).

TAT was measured to detect coagulation activation (Figure 5A). Contact of blood with untreated PCU resulted in a significant increase in TAT levels (*p* ≤ 0.001, compared to control blood). The TAT level of untreated PCU was 3.4 times higher than that of PEG-DEX-AT (2.1/11.1) (*p* = 0.012), and 3.7 times higher than that of PEI-AT (3.2/23.4) (*p* = 0.002). Levels of the complement factor SC5b-9 significantly increased in the presence of PEG-DEX-AT (*p* ≤ 0.001) (Figure 5B). Untreated PCU and PEI-AT had no significant effect on complement activation. Platelet hyperactivity was measured by the release of ß-TG (Figure 5C). Untreated PCU and PEG-DEX-AT caused a significant release of ß-TG (*p* ≤ 0.001; *p* ≤ 0.001 compared to negative control), but PEI-AT did not induce platelet activation (*p* = 0.346). The protective effect of PEI-AT was 2.4 times lower than that of untreated PCU (1.3/6.9) (*p* ≤ 0.001). These data correlated with the number of platelets (Figure 5D). Blood contact with untreated PCU and PEG-DEX-AT significantly decreased platelet counts (*p* = 0.007; *p* = 0.003 compared to negative control). No change in platelet count was observed for PEI-AT (*p* = 0.316). There was no difference between the samples. Granulocyte activation was measured by means of the release of PMN-elastase (Figure 5E). Inflammatory response was highest for PEG-DEX-AT (*p* ≤ 0.001). Untreated PCU and PEI-AT had no significant effect on leukocyte activation.

## 4. Discussion

Chemical modifications have been used to improve the hemocompatibility of Chronoflex AR as a potential material for the pump chamber of implantable VAD systems [20]. Among the modified PCU samples used in the present study, PEI-AT modification had minor effects on mechanical properties and showed increased wettability, reduced bacterial cell binding, suppressed platelet adhesion and activation, and activation of complement and coagulation under shear stress.

Plasma treatment and chemical modification alter the physical and thermal properties of Chronoflex AR. Each modification employed in our study decreased the glass transition temperature of PCU, which was attributed to a greater flexibility of the modified PCU discs [27,28]. The two modifications did not differ with regard to their elastic properties.

Each surface modification significantly reduced the adhesion and proliferation of *S. epidermidis* to untreated PCU samples. The introduction of PEG or PEI-based hydrogels may be responsible for preventing bacterial adhesion [29,30]. Park et al. [29] used different PEG modifications on polyurethane surfaces and showed a significant reduction in *S. epidermidis* adhesion on sulfonated PEG, long-chain PEG, and heparinized PEG surfaces. Such coatings show antifouling properties and significantly reduce protein adsorption and bacterial adhesion because of the high entropy and strong affinity of PEG for water [31]. Furthermore, coating glass or polyethylene slides with hydrophobic *N*-alkyl-PEI polycations kills gram-positive human pathogen Staphylococcus aureus and gram-negative *Escherichia coli* [32]. These surface modifications may be useful to reduce bacterial colonization on medical devices. Clinical data have reflected the necessity of such antimicrobial surface coatings. Rosenfeldt et al. [33] showed in a single center study that 45% of patients with a VAD developed VAD-associated bacteremia with an incidence of 5.6 episodes per 1000 support days.

The AT-modified samples in the current study resisted platelet adhesion and activation, which was impressively shown by SEM. Under static conditions, activated platelets accumulate on untreated PCU surfaces and produce extensive fiber networks. The inadequate hemocompatibility of untreated polyurethanes was approved by other studies [2,14,34], limiting its use in blood-contacting applications. We supposed that the hydrophobic character of untreated PCU caused a higher adhesion of platelets. The immobilization of PEG-DEX-AT and PEI-AT modifications reduced hydrophobicity of the surface, prevented platelet adhesion and suppressed the outspread and pseudopodium deformation of the adherent platelet, which indicates improved blood compatibility. In a previous study, our group demonstrated that the binding of PEG-DEX and PEI hydrogels to plasma-activated PCU significantly reduced platelet adhesion under static conditions [20]. This was confirmed by Coll Ferrer et al. [35]. The additional immobilization of AT did not improve the thromboresistance of the material [20]. However, it could not be excluded that the immobilized AT performed its physiological function. Similar results were shown by Ito et al. [36], who synthesized an acrylamide derivative of a thrombin inhibitor and polymerized it to the surfaces of polymer membranes. This construct deactivated thrombin and suppressed the adhesion of platelets. Furthermore, surface coating with argatroban, a direct thrombin inhibitor, reduced thrombus formation in an extracorporeal circulation circuit [15].

Finally, the Chandler-Loop model was used in consideration of the fact that blood-contacting devices not only interact with blood in direct proximity but also with the entire circulating blood volume [37,38]. The Chandler-Loop model simulates arterial flow conditions [39,40] and enables the testing of hemocompatibility of artificial materials according to ISO 10993-4 standard tests [41]. The limited hemocompatibility of polyurethane was shown by the activation of the coagulation cascade exemplified by an increase in TAT and ß-TG levels and a decrease in platelet count, whereas the complement and inflammatory systems were only slightly influenced. Alterations in the hemostatic response were inhibited in contact of PCU with immobilized AT. However, only the PEI-AT modification significantly reduced coagulation (TAT) and platelet activation (ß-TG) without altering the complement and granulocyte reaction. PEG-DEX-AT modification only reduced the levels of TAT; platelet activation remained high, complement and inflammatory systems were stimulated, and platelets adhered to the inner surfaces of the modified tubular grafts.

With the Chandler-Loop model, we were able to compare more than two different PCU surface modifications in one experimental setting, which is a major limitation of other in vitro as well as in vivo models. Nevertheless, one limitation of our study was the limited surface characterization of the modified PCU discs (FTIR, NMR) and missing data from intermediate modified PCU samples. More details have already been published [20]. Furthermore, mechanistic studies on the functionality of AT failed. However, the present study was focused on the biological responses of different surface modifications to human blood cells under shear stress. Further studies will be necessary for additional characterization, particularly of the surface properties.

## 5. Conclusions

While both PEG-DEX-AT and PEI-AT significantly reduce coagulation activation, only PEI-AT creates anti-bacterial and anti-thrombogenic functionality. Future studies will show whether this modification is suitable for cardiovascular tissue engineering.
